# The impact of postoperative atrial fibrillation on complications and mortality following Ivor Lewis esophagectomy for esophageal cancer

**DOI:** 10.1038/s41598-025-06239-8

**Published:** 2025-07-01

**Authors:** Saeed Torabi, Philipp Omuro, Fabian Dusse, Naita M. Wirsik, Thomas Schmidt, Wolfgang Schröder, Tobias Kammerer, Andrea U. Steinbicker, Hans A. Schlößer, Christiane J. Bruns, Hans F. Fuchs, Lars M. Schiffmann

**Affiliations:** 1 Department of Anesthesiology and Intensive Care Medicine, Medical Faculty of Cologne University, University Hospital of Cologne, Cologne, Germany; 2 Department of General, Visceral, Thoracic and Transplant Surgery, Medical Faculty of Cologne University, University Hospital of Cologne, Cologne, Germany

**Keywords:** Postoperative atrial fibrillation, Ivor-Lewis esophagectomy, Anastomotic leakage, Pulmonary complications, Postoperative morbidity, Oesophageal cancer, Gastrointestinal cancer

## Abstract

Postoperative atrial fibrillation (POAF) is a common complication following transthoracic esophagectomy, with an incidence rate between 12% and 37%. POAF has been associated with adverse outcomes, including pulmonary complications, anastomotic leakage, prolonged ICU stays and increased in-hospital mortality. This study investigates the impact of POAF on postoperative outcomes in a high-volume center and evaluates its role as a predictor of postoperative complications following Ivor-Lewis esophagectomy.This retrospective, single-center cohort study was conducted at the University Hospital of Cologne, Germany. Patients, who underwent elective Ivor-Lewis esophagectomy for cancer, were included (*n* = 617). Perioperative data were extracted retrospectively from a prospectively maintained database, capturing demographic, surgical, and postoperative variables. POAF was defined as a hemodynamically significant tachyarrhythmia absoluta that occurs within the first 7 days postoperatively and has been confirmed via ECG. Statistical analyses included univariate and multivariate logistic regression to identify associations between POAF and postoperative outcomes.POAF occurred in a significant proportion of patients (*n* = 79, 12,8%) and was strongly associated with adverse outcomes. Patients with POAF demonstrated higher rates of pulmonary complications (24.0% vs. 11.2%, *p < .01), anastomotic leakage (32.9% vs. 10.5%, *p < .01), and prolonged ICU stays (median 7 days vs. 2 days, *p < .01). The in-hospital mortality rate in the POAF group was 7.6%, compared to 1% in patients without POAF (*p < .01). Independent predictors of POAF included older age, pre-existing atrial fibrillation and beta-blocker therapy. Postoperative atrial fibrillation (POAF) was a significant predictor of adverse postoperative outcomes. Logistic regression analysis revealed that POAF was associated with higher odds of anastomotic leakage (OR = 3.11, *p < .01), ICU readmission (OR = 6.80, *p < .01), in-hospital mortality (OR = 6.76, *p < .01) and 90-Day mortality ( OR = 5.44, *p < .01). In our cohort, POAF was not significantly associated with oncological recurrence (OR = 0.71; *p* = .219). These findings highlight the critical role of POAF in predicting postoperative complications.POAF is both - a complication and a potential marker - for systemic stress, predicting further adverse events such as anastomotic leakage and respiratory insufficiency. Although some studies suggest that POAF does not affect long-term survival, its pronounced impact on short-term morbidity underscores the necessity of early identification and focused management. Preoperative risk stratification and intraoperative strategies, such as goal-directed fluid therapy, could mitigate the impact of POAF. Postoperative atrial fibrillation (POAF) significantly influences recovery after esophagectomy, serving as a marker for increased morbidity and mortality. Advanced age, hypertension, and respiratory complications emerged as key independent risk factors. Additionally, POAF was linked to longer hospital stays, in-hospital and 90-day mortality and a heightened incidence of postoperative complications, including pneumonia and anastomotic leakage. These results highlight the critical importance of implementing targeted perioperative strategies to reduce risks and enhance outcomes in this high-risk patient population.

**Supplementary Information:**

The online version contains supplementary material available at 10.1038/s41598-025-06239-8.

## Introduction

Esophagectomy is the cornerstone in multimodal treatment of esophageal cancer and offers the potential for curative resection^1^. Despite significant advancements in surgical techniques and perioperative care, this procedure remains associated with a substantial risk of postoperative complications^1,2^. Among these, postoperative atrial fibrillation (POAF) is one of the most common and clinically significant arrhythmias^2^ with an incidence reported to range between 12% and 37% of cases depending on patients` characteristics and the surgical approach^3,4^​.

The occurrence of POAF has been linked to a range of adverse outcomes^1,4^ including respiratory failure, prolonged intensive care unit (ICU) stay, and elevated postoperative mortality rates^1,4^. These associations highlight the critical importance to understand and effectively manage POAF in the context of esophagectomy.

Recent studies suggest that POAF may also serve as an early clinical marker for serious complications, such as pulmonary complications and anastomotic leakage, both of which can profoundly impact recovery and overall prognosis^5,6^. Pulmonary complications, including pneumonia and respiratory insufficiency, have been frequently reported in patients with POAF, often leading to extended ICU stays and delayed discharge^5–8^. Anastomotic leakage, a potentially life-threatening complication is particularly concerning^9^. POAF has been associated with significant morbidity, requiring invasive interventions, prolonged hospitalization^7,9,10^​.

The potential role of POAF as both, a complication^2^ and a predictor^5,6^ of further adverse events following esophagectomy warrants comprehensive investigation. Specifically, it raises questions about the pathophysiological mechanisms linking POAF with complications such as anastomotic leakage^11^ as well as the utility of POAF as a prognostic marker in postoperative care. By identifying at-risk patients and early therapy^12^ clinicians may be able to mitigate the downstream effects of POAF and improve patients` outcome.

This study seeks to evaluate the impact of POAF on the postoperative course in patients undergoing Ivor-Lewis esophagectomy for cancer. Particular attention will be paid to the incidence of pulmonary complications, the lengths of stay at the ICU, postoperative mortality and the occurrence of anastomotic leakage. To elucidate the relationships between POAF and these outcomes, this research aims to enhance risk stratification and inform targeted strategies to optimize perioperative care in esophagectomy patients.

## Methods

### Study design and setting

This retrospective, single-center cohort study was conducted at the Department of Anesthesiology and Intensive Care Medicine and the Department of General, Visceral and Transplant Surgery, Faculty of Medicine, University Hospital of Cologne, University of Cologne, Cologne, Germany. All patients included in this study underwent surgery at the Department of General, Visceral and Cancer Surgery and subsequently received postoperative intensive care treatment at the intensive care units of the Department of Anesthesiology and Intensive Care Medicine.

### Patient selection

Patients, who underwent elective Ivor-Lewis esophagectomy, characterized by laparoscopic or open gastrolysis and open thoracic or minimally invasive thoracoscopic transthoracic esophagectomy, were included. Exclusion criteria were:

- Emergency esophagectomy for trauma or Boerhaave syndrome.
- McKeown esophagectomy with cervical anastomosis.
- benign indications such as achalasia.

This selection ensured a homogeneous patient cohort undergoing a standardized surgical approach (open, hybrid or total minimally invasive Ivor-Lewis esophagectomy) and postoperative critical care.

### Data collection

Perioperative data were retrospectively collected from a institutional database. Electronic data sources included hospital systems such as Orbis, while paper-based documentation from ICU charts, patient files, and anesthesia records was reviewed.

Data extraction included:

- Demographic data: Age, sex, and relevant comorbidities.
- Surgical variables: Surgical approach (Ivor-Lewis hybrid), operative duration, and intraoperative parameters.
- Postoperative outcomes: Incidence of hemodynamically significant postoperative tachyarrhythmia absoluta (confirmed by ECG), pulmonary complications, anastomotic leakage, ICU length of stay, and in-hospital mortality.

Definitions:

Postoperative atrial fibrillation (POAF) was defined as a hemodynamically significant postoperative tachyarrhythmia absoluta occurring within the first 7 days after surgery. Day of surgery is defined as day zero. The diagnosis was confirmed via electrocardiogram (ECG) and required either medical or electrical intervention within this period.

After ruling out potential underlying causes of postoperative atrial fibrillation (POAF), such as hypovolemia or electrolyte imbalances, further diagnostics are conducted using esophagogastroduodenoscopy (EGD) and/or computed tomography (CT) to exclude anastomotic leakage or pneumonia. If these complications are ruled out, POAF is initially managed according to guidelines with beta-blockers for rate control^13^. Subsequently, anticoagulation therapy is initiated following the recommendations of the American Association for Thoracic Surgery (AATS) to reduce the risk of thromboembolic complications associated with atrial fibrillation^14,15^.

Postoperative pulmonary complications were defined as the occurrence of pneumonia, clinically significant atelectasis requiring non-invasive ventilation (NIV) therapy, or pleural effusions that require placement of a chest tube. These complications were further characterized by the presence of either partial or global respiratory insufficiency.

### Ethical approval

This study is in accordance with the ethical standards of the institutional research committee and with the 1964 Helsinki declaration and its later amendments or comparable ethical standards. The study protocol was approved by the institutional ethics committee of the University Hospital of Cologne (Approval No. 24-1319). As this study was conducted retrospectively, patients did not have to sign an informed consent, and all patients` data were anonymized for analyses.

### Statistical analysis

Data was obtained from the electronic patient data management system (PDMS) and analog documents items were imported anonymously in Microsoft^®^ Excel V. 16.77.1 (Microsoft^®^, Redmond, WA, USA). For statistical analysis data was transferred to IBM SPSS V. 29.0.2.0 (20) (IBM, Armonk, NY, USA). Baseline characteristics and perioperative outcomes were summarized using appropriate descriptive statistics. Continuous variables were expressed as means with 25.-75. percentiles. Binary variables are presented as frequencies and percentages. Comparisons between groups were performed using the χ^2^ (categorial variables) and Mann-Whitney-U (continuous variables). A p-value of < 0.05 was considered statistically significant for all analyses.

## Results

This retrospective cohort study included 617 patients who underwent a minimally invasive hybrid Ivor Lewis esophagectomy between June 2016 and June 2023 at University Hospital of Cologne. All patients were treated for esophageal cancer and underwent surgery as part of their curative treatment plan. There were no significant differences regarding the duration of the surgery (median duration was 6.0 h in both groups, *p* > .05) and the duration of one-lung ventilation (OLV) in patients with or without POAF.

Of the 617 patients, 79 (12.8%) developed POAF postoperatively. Patients with POAF were significantly older (67 [62–73] vs. 62 [56–69] years, *p* < .01. The median age of the study population was 63 [56–70] years (Table 1), with a considerable proportion of patients presenting pre-existing conditions such as arterial hypertension (*n* = 355, 57.5%), diabetes mellitus (*n* = 103 16.7%), heart failure (*n* = 101, 16.4%) and chronic obstructive pulmonary disease (*n* = 77, 12.5%). Preoperative atrial fibrillation was observed in 9.6% of the patients (Table 2).

Cardiovascular risk factors were more frequent in patients with POAF vs. non-POAF, such as arterial hypertension (68% vs. 58%, *p* < .01), history of myocardial infarction (20% vs. 10%, *p* = .04), preexisting atrial fibrillation (26% vs. 12%, *p* = .01) and Diabetes mellitus (27% vs. 15%, *p* = .03).

In the multivariate logistic regression analysis age emerged as the only significant predictor of postoperative atrial fibrillation (POAF). The regression coefficient was B = 0.11, with an odds ratio (Exp(B)) of 1.12 and a corresponding p-value of 0.02, indicating that the risk of developing POAF increases by approximately 12% with each additional year of age.

In contrast, other variables included in the analysis—arterial hypertension, history of myocardial infarction, preexisting atrial fibrillation and diabetes mellitus did not demonstrate statistically significant associations with POAF.

- Arterial hypertension showed a weak positive association (B = 0.09; Exp(B) = 1.10), which was not statistically significant (*p* > .05).
- A previous myocardial infarction was associated with a higher odds ratio of 3.07 (B = 1.12), yet this result did not reach significance (*p* > .05).
- Preexisting atrial fibrillation yielded a negative regression coefficient (B = -1.64) and an odds ratio of 0.19, indicating a potential inverse relationship with POAF—i.e., patients with known atrial fibrillation prior to surgery may have a lower likelihood of developing new-onset POAF postoperatively. However, the association was not statistically significant (*p* > .05), and thus the finding must be interpreted with caution.
- For diabetes mellitus, the regression coefficient was B = -0.05, with an odds ratio of 0.96 and *p* > .05, suggesting a non-significant 4% decrease in POAF risk—again, statistically unsupported association.

**Table 1:** Patient baseline characteristics. [units]. Mean [25.-75. Percentile]. *BMI* Body mass [units].dex, *ASA* American society of anesthesiology.

Age [years]	63.0 [56.0–70.0]
Height [cm]	178 [171–183]
Weight [kg]	83.0 [72.0–83.0]
BMI [kg/m^2^]	26.45 [23.65–29.86]
ASA-Score	2 [2–3]

**Table 2:** Preoperative medical history. % [absolute values]. *CABG* cardiac artery bypass grafting. *COPD* chronic obstructive pulmonary disease. *PAD* peripheral artery disease.

Arterial hypertension	57.5% [355]
CABG	2.4% [15]
Heart failure	16.4% [101]
Myocardial infarction	7.5% [46]
Atrial fibrillation	9.6% [59]
COPD	12.5% [77]
Diabetes mellitus	16.7% [103]
PAD	4.4% [27]
Stenosis of coeliac trunk	3.6% [22]
Stenosis of superior mesenteric artery	1.3% [8]
Abuse of Nicotine	65.2% [402]
Abuse of Alcohol	19.1% [118]

We identified significant associations between POAF and several adverse postoperative outcomes, including pulmonary complications, anastomotic leakage, extended ICU stays, and increased short-term mortality. Postoperatively, 17.7% of patients experienced pulmonary complications, with pneumonia being the most common (12.8%). Anastomotic leakage occurred in 12.5% of patients, and 6.8% required prolonged mechanical ventilation for more than 72 h. The in-hospital mortality rate was 1.8%, and ICU readmissions occurred in 12.8% of cases, with a median ICU stay of two days (Tables 3 and 4).

**Table 3:** Postoperative complications following Ivor-Lewis Esophagectomy. % [absolute values]. *POAF* Postoperative atrial fibrillation *ARDS* acute respiratory distress syndrome. *ICU* intensive care unit. *h* hours.

Enterothorax	1.3% [3]
Delirium	5.8% [36]
Reintubation	10.7% [66]
Tracheoesophageal fistula	0.2% [1]
Pneumonia	12.8% [79]
Pylorospasm	24.1% [149]
Recurrent nerve injury	2.3% [14]
Anastomic leakage	12.5% [77]
Conduit necrosis	1.8% [11]
Mortality (intrahospital)	1.8% [11]
ARDS	1.6% [10]
Chylothorax	1.5% [9]
ICU readmission	12.8% [79]
Tachyarrhythmia absoluta	17.7% [109]
Atelectasis with need for bronchoscopy	2.9% [18]
Tracheotomy	4.4% [27]
Respiratory failure with mechanical ventilation > 72 h	6.8% [42]

**Table 4:** Comparison of patient with and without postoperative atrial fibrillation (POAF). [units]. Mean [25.-75. percentile]. % [absolute values]. * = [units].gnificant [units]. *p* < .05.

	POAF (*n* = 79)	Non-POAF (*n* = 538)	Test- and *p*-values
Anastomotic leakage	32.9% [26]	10.5% [51]	χ^2^ = 34.6; *p* < .01*
Pneumonia	24.0% [19]	11.2% [60]	χ^2^ = 10.3; *p* < .01*
Tracheotomy	11.4% [9]	3.3% [18]	χ^2^ = 10.7; *p* < .01*
Mechanical ventilation > 72 h	16.5% [13]	5% [29]	χ^2^ = 13.3; *p* < .01*
ICU-readmission	35.4% [28]	10.2% [55]	χ^2^ = 37.6; *p* < .01*
Arterial Hypertension	65.6% [52]	57.9% [303]	χ^2^ = 8.8; *p* = .04 *
Preexisting atrial fibrillation	23.5% [12]	12.4% [47]	χ^2^ = 4.7; *p* < .05*
Beta-Blocker	59.7% [46]	33.7% [179]	χ^2^ = 19.5; *p* < .01*
Age	67 [62–73]	62 [56–69]	U = 27926.0; *p* < .01*
Length of ICU stay	7 [3–13]	2 [2–4]	U = 33191.5; *p* < .01*
In-hospital Mortality	7.6% [6]	1% [5]	χ^2^ = 17.48; *p* < .01*

Patients who developed POAF, demonstrated worse outcomes compared to those without POAF (Table 4). Anastomotic leakage was markedly more frequent in the POAF group (32.9% versus 10.5%, χ^2^ = 34.6; *p < .01), and pneumonia rates were higher (24.0% versus 11.2%, χ^2^ = 10.3; *p* < .01*). Prolonged mechanical ventilation was also more common in the POAF group (16.5% versus 5%, χ^2^ = 10.3; *p* < .01). Additionally, ICU readmissions were significantly more frequent among patients with POAF (35.4% versus 10.2%, χ^2^ = 37.6; *p < .01), and these patients had a longer ICU stay (median of 7 days compared to 2 days, U = 33191.5; *p < .01).

The in-hospital mortality rate was significantly higher in the POAF group at 7.6%, compared to 1% in those without POAF (χ^2^ = 17.48; *p < .01) (Table 4). Further analysis revealed a significantly increased 90-day mortality in patients who developed POAF (7.0%) compared to those without POAF (1.4%) (χ² = 9.266, *p* = .011), The calculated odds ratio (OR) was 5.44, indicating that patients with POAF had more than a fivefold increased odds of dying within 90 days postoperatively compared to those without POAF. These findings suggest that POAF may serve not only as a complication but also as a valuable early marker of postoperative mortality risk.

We further examined the association between POAF and oncological recurrence. Among patients with POAF, the recurrence rate was 32.3%, compared to 40.4% in patients without POAF. However, the difference did not reach statistical significance (χ² = 1.510, *p* = .219). These findings suggest that POAF was not significantly associated with tumor recurrence in our cohort.

Patients with POAF tended to be older (median age 67 versus 62 years) and had a higher prevalence of pre-existing atrial fibrillation (23.5% versus 12.4%). Furthermore, beta-blocker therapy prior to surgery was more common among patients who developed POAF (59.7% versus 33.7%, χ^2^ = 19.5; *p* < .01).

Given the significant impact of POAF on outcomes, it is important to investigate whether POAF occurs spontaneously, contributes to the development of complications, or is itself a consequence of these complications.

Binary logistic regression analysis demonstrated that POAF significantly predicted anastomotic leakage, ICU readmission, and in-hospital mortality.

In binary logistic regression, POAF predicted anastomotic leakage (B 1.13, r 0.33, Wald 11.83, df = 1, Exp(B) 3.11, *p* < .01), ICU-readmission (B 1.10, r 0.32, Wald 10.44, df = 1, Exp(B) 6.80, *p* < .01) and in-hospital mortality (B 1.91, r 0.72, Wald 7.00, df = 1, Exp(B) 6.76, *p* < .01).

Specifically, POAF was associated with increased odds of anastomotic leakage (B = 1.13, *r* = .33, Wald χ²(1) = 11.83, Exp(B) = 3.11, *p* < .01), indicated that the likelihood of anastomotic leakage was approximately three times higher in patients with POAF.

Similarly, POAF was a significant predictor of ICU readmission (B = 1.10, *r* = .32, Wald χ²(1) = 10.44, Exp(B) = 6.80, *p* < .01), with a nearly sevenfold increased risk of ICU readmission in affected patients.

In addition, POAF strongly predicted in-hospital mortality (B = 1.91, *r* = .72, Wald χ²(1) = 7.00, Exp(B) = 6.76, *p* < .01), corresponding to an approximately sevenfold increase in the odds of mortality.

## Discussion

POAF is among the most frequent and clinically significant complications following esophagectomy, with a reported incidence ranging from 10 to 40% depending on surgical technique^16–18^ patients demographics, and perioperative factors​^2,4^. Our results demonstrate that POAF not only represents a direct postoperative challenge but also serves as a marker and possible predictor for subsequent complications such as pulmonary complications, anastomotic leakage, extended ICU stays and increased in-hospital mortality.

Our findings corroborate those of prior studies highlighting the association between POAF and postoperative pulmonary complications, including pneumonia and respiratory insufficiency^4,19^. This observation is in line with the observations of Seesing et al. reporting that POAF is strongly associated with infectious pulmonary complications such as pneumonia^3^​. Murthy et al. identified a similar relationship with an increase of POAF and the likelihood of pulmonary issues and systemic inflammatory responses that exacerbate postoperative morbidity​^10^ .

Anastomotic leakage was another major complication strongly associated with POAF in our cohort, reflecting previous findings by Feng et al., who noted a heightened risk of anastomotic failure in patients with arrhythmias^20^. POAF may serve as an early clinical indicator of complications such as anastomotic leakage, as suggested by studies that demonstrate the temporal relationship between POAF onset and subsequent complications^5,7,10,21^. The exact mechanisms underlying the association of arrhythmias, POAF and anastomotic leakage likely involve hemodynamic instability and reduced tissue perfusion, which impair healing of surgical anastomosis, further emphasizing the need for close monitoring of patients hemodynamic intra- but also postoperativly^11^.

The occurrence of POAF following esophagectomy is a multifactorial process driven by a combination of surgical, patient-specific, and systemic factors^2^. Direct surgical trauma^17,18^inflammation^22^ fluid shifts, electrolyte imbalance and autonomic dysregulation of the nervous system are key contributors^3,4^​. Surgical trauma triggers a systemic inflammatory response, marked by elevated levels of pro-inflammatory markers. This inflammatory state contributes to atrial remodeling and enhances atrial ectopic activity, thereby increasing susceptibility to atrial fibrillation^23,24^. The inflammatory burden may be further amplified by postoperative complications, including pneumonia and anastomotic leakage, both of which have been strongly associated with the development of POAF^7,10^.

The strong association between POAF and factors such as advanced age, hypertension, and the Ivor-Lewis surgical approach observed in our study aligns with previously identified risk factors reported in the literature^2,8,17,25^. Additionally, Kashiwagi et al. highlighted the impact of surgical complexity, such as extended lymph adenectomy, the anatomy of pulmonary veins and left atrial size as an additional risk factor^26,27^​. From this perspective, the work of Nagatsuka et al.^6^ is particularly intriguing and underscores the importance of preoperative echocardiographic evaluation. Preoperative diagnostic procedures, often performed as part of staging or perioperative planning, such as thoracic and abdominal CT scans, can also be specifically evaluated with regard to anatomical conditions. Such an approach could help to reduce the incidence of postoperative atrial fibrillation and its associated complications as part of comprehensive risk stratification efforts.

While these mechanisms are well-supported, the pathogenesis of POAF remains incompletely understood. It has been proposed that single-lung ventilation and perioperative oxidative stress may lead to mitochondrial dysfunction in cardiac tissue, precipitating arrhythmias^3,4^​. This underscores the need for perioperative strategies with the aim to minimize inflammatory and oxidative stress responses^22^.

There are various perspectives of the association between POAF and adverse postoperative outcomes. On one hand, POAF might be the cause for further complications, on the other hand, POAF might also be the symptom for various underlying issues. Due to the potentially life-threatening complications associated with POAF, often signaled by the new onset of tachyarrhythmia, we consider postoperative atrial fibrillation primary as an early alarm sign for underlying causes such as anastomotic leakage. Therefore, after ruling out factors such as volume deficits, electrolyte imbalances, or reactive tachycardia due to the discontinuation of preoperative beta-blocker therapy, we proceed with further diagnostic evaluations. These include esophagogastroduodenoscopy (EGD) and thoracic CT scans to exclude the possibility of anastomotic leakage. Therefore, we critically question the recommendation by Ojima et al.^28^ and Tisdale et al.^29^. who suggest the preemptive administration of Landiolol or Amiodaron to prevent POAF. Although, prophylactic administration of landiolol has been demonstrated to reduce the incidence of POAF and its associated complications, such as anastomotic leakage and prolonged ICU stays^28,30^​. The prophylactic administration of antiarrhythmic agents could potentially lead to the masking of the resulting tachyarrhythmia, thereby delaying the diagnosis of a potentially far-reaching complication such as an anastomotic leak. If other potential causes for the occurrence of POAF have been ruled out, the tachyarrhythmia can be managed in accordance with the guidelines for the treatment of atrial fibrillation^31^.

Management of postoperative atrial fibrillation (POAF) after esophagectomy prioritizes rate control and anticoagulation^13,32^. Beta blockers are recommended first-line by the ACC/AHA, with calcium channel blockers as alternatives. Amiodarone is reserved for cases where other therapies fail or are contraindicated^13,32^. for anticoagulation, guidelines suggest continuing therapy for 4 weeks post-return to sinus rhythm to mitigate thromboembolism risks from impaired atrial contraction, with the choice of anticoagulant tailored to the patient’s clinical profile^14,15^. This approach ensures optimized cardiovascular stability and minimizes complications in the postoperative period.

Our findings support the hypothesis that POAF serves not merely as a complication but as a predictor for subsequent adverse events. Similarly, the findings of Stawicki et al.^7^ and Seesing et al.^3,5^ highlight POAF as an early warning sign and predictor of further postoperative complications. Stawicki et al. also emphasized the increased mortality associated with the onset of POAF and additionally questioned the efficacy of prophylactic therapy for atrial fibrillation​^7^.

While some research, such as an article by Ojima et al. suggests that POAF does not significantly affect long-term survival^33^ its role in short-term morbidity and mortality cannot be understated​^4,7,10,19^. In our cohort, patients with POAF exhibited an increased mortality rate of 7.6%, which appears comparable to findings from other groups^7^ that reported reduced 60-day survival rates in patients with POAF^10,16,34^.

The temporal association of POAF with other complications underscores the importance of early identification and focused therapy to prevent cascading clinical deterioration^1,8,35^. POAF can ultimately be viewed as a consequence of various factors, including volume or electrolyte imbalances, pre-existing atrial fibrillation, prior beta-blocker therapy^26,36^ or intraoperative surgical manipulation of the pericardium^17,18^. However, it may also serve as an early indicator of further postoperative complications^7,21^ such as anastomotic leakage. This multifactorial etiology underscores the complexity of POAF and its potential role in the postoperative course.

Recognizing POAF as a significant marker for postoperative risk carries several outcome-relevant implications for clinical practice. Preoperative risk stratification, particularly in older patients with comorbidities, should be prioritized to identify those at higher risk^36^. Intraoperative strategies such as goal-directed fluid therapy (GDT) have shown promising results to reduce pulmonary complications and to maintain hemodynamic stability ​^37^. Postoperatively, close hemodynamic monitoring for the early detection of POAF is crucial. It must also be emphasized that preoperative evaluation as part of risk stratification, combined with effective intraoperative management, can significantly improve postoperative outcomes and reduce mortality. This is supported by the findings of Bartels et al.^36^ who demonstrated that preoperative risk analysis reduced lethality from 9.4 to 1.6%.

Based on our findings, we propose a multimodal perioperative approach to reduce the risk of POAF, emphasizing individualized care and early recognition of complications. Key components include preoperative risk stratification based on age, inflammatory markers, and cardiac comorbidities; continuation of beta-blockers when indicated; and the maintenance of intraoperative hemodynamic stability. While prophylactic antiarrhythmic therapy with agents such as Landiolol or Amiodarone has shown efficacy, its routine use should be approached cautiously. Early administration may mask warning signs of serious complications like anastomotic leakage. Therefore, prophylaxis should be reserved for carefully selected high-risk patients, ensuring that arrhythmia prevention does not delay the diagnosis of underlying surgical issues.

In this context, the development of a risk score incorporating relevant preoperative and intraoperative risk factors could be a promising approach to improve patient outcomes and identify those at higher risk. Although our study provides valuable insights, it is important to acknowledge its retrospective design, which limits causal interpretations. Further exploration of the mechanistic pathways linking POAF to complications like anastomotic leakage and pulmonary complications is also warranted.

## Conclusion

This study demonstrates that newly occurred POAF significantly impacts outcome after esophagectomy, being strongly associated with adverse outcomes such as pulmonary complications, anastomotic leakage, prolonged ICU- stays and increase in- hospital and 90-day mortality. Patients with POAF face disproportionately higher rates of complications, suggesting that POAF is not only a complication, but also serves as a clinical warning sign for a complicated postoperative course.

Given the association of POAF with predisposing factors such as older age, pre-existing atrial fibrillation, and beta-blocker therapy, targeted preoperative risk stratification and optimized management strategies are essential. Early detection and prompt treatment of POAF could mitigate its downstream complications.

## Author contributions

S.T. and L.S. designed the study. P.O. performed the formal statistical analysis of the data provided by L.S., N.W. and W.S. S.T, P.O., F.D. interpreted the data. S.T. wrote the inaugural draft and final draft of the manuscript. A.S. and T.K. critically revised A.S. and L.S. revised the final manuscript and substantially supervised the manuscript. C.B., H.S, H.F. und L.S. contributed to the study concept and supervision. All authors approved the final submitted version of the manuscript.

## Electronic supplementary material

Below is the link to the electronic supplementary material.

Supplementary Material 1

Supplementary Material 2

Supplementary Material 3

## Data Availability

The datasets used and analyzed during the current study are available from the corresponding author upon reasonable request.
