# Supplementary material for: The impact of postoperative atrial fibrillation on complications and mortality following Ivor Lewis esophagectomy for esophageal cancer
Source: Sci Rep. 2025 Jul 1;15:22305. doi: 10.1038/s41598-025-06239-8 (PMC12217222; doi:10.1038/s41598-025-06239-8)
Supplement: Supplementary file 1 — Supplementary Material 1 [file 41598_2025_6239_MOESM1_ESM.pdf]

ige of authorship request form - Journals (pre-acceptance)

(1)

ithorship (adding/deleting authors) including changes in corresponding authors. This form should not be used for
nd block capitals and provide each author's full name with the given name first followed by the family name.

of the authors are in accordance with their scientific contribution, if applicable as different conventions apply per
neaningful contribution to the work.

ement for representing the collaboration, it is sufficient for the representative or legal guarantor (usually the
nge Form on behalf of all authors, **next to the added/removed author(s)**. **(Complete Section 3, followed by Section**

representing the collaboration and **there are more than 10 authors**, one may sign for all, provided the signer
ave agreed to the change **and the added/removed authors sign the form**. **(Complete Section 3, followed by Section**

sputes. If you are unable to obtain agreement from all authors (including those who you wish to be removed) you
ase inform us if you need to do this.

ys of the date that it was sent to the author requesting the change, we may have to withdraw your manuscript. We
ed by all authors (including those who have been removed).

Editorial Office. The Journal and/or Publisher will consider the information you have provided to decide whether to
contact your institution for more information or undertake a further investigation, if appropriate, before making a

irch, educational and professional publishers, created in May 2015
ation and Springer Science+Business Media.

ıge of authorship request form - Journals (pre-acceptance)

(2)

Manuscript ID no.: e2e6f9c9-847e-4da4-a1e7-9b8798225dcc v2.0

ɔhagectomy: A predictor for Complications and Mortality in Esophageal Cancer Surgery -

n the manuscript before the changes were introduced. Please indicate the corresponding author by adding (CA)

ıame	ORCID or SCOPUS id, if available
:	
rer	
ɜr	0000-0002-5237-961X

arch, educational and professional publishers, created in May 2015
:ation and Springer Science+Business Media.

ge of authorship request form - Journals (pre-acceptance)

(3)

Manuscript ID no.:

n the manuscript before the changes were introduced. Please indicate the corresponding author by adding (CA)

ame	ORCID or SCOPUS id, if available
ann	

arch, educational and professional publishers, created in May 2015
ation and Springer Science+Business Media.

(4)

ıge of authorship request form - Journals (pre-acceptance)

on to explain your reasons for changing the authorship of your manuscript, e.g. what necessitated the change in :ion about authorship. Please explain why omitted authors were not originally included and/or why authors were

r has been added to the author list. As the former head of esophageal surgery and initiator of the port in shaping the study concept and made significant contributions to the manuscript, especially

nts during the submission process. All authors, including Professor Schröder, have reviewed and change in authorship.

ip list in the order you would like it to appear on the manuscript. Please indicate the corresponding author by **, please indicate the reason under section 3.**

ear in full on the final in various abstract	Affiliated institute	E-mail address
		saeed.torabi@uk-koeln.de
		philipp.omuro@uk-koeln.de
		fabina.dusse@uk-koeln.de
		Naita.wirsik@uk-koeln.de
		thomas.schmidt1@uk-koeln.de
		Wolfgang.schröder@helios-gesundheit.de
	Dept. of Anesthesiology & ICM	tobias.kammerer@uk-koeln.de
	Dept. of Anesthesiology & Intensive Care Medicine	andrea.steinbicker@uk-koeln.de
		hans.schlößer@uk-koeln.de
		christiane.bruns@uk-koeln.de

ırch, educational and professional publishers, created in May 2015
ation and Springer Science+Business Media.

ıge of authorship request form - Journals (pre-acceptance)

(5)

ion to explain your reasons for changing the authorship of your manuscript, e.g. what necessitated the change in tion about authorship. Please explain why omitted authors were not originally included and/or why authors were

ıip list in the order you would like it to appear on the manuscript. Please indicate the corresponding author by
l, please indicate the reason under section 3.

ear in full on the final : in various abstract	Affiliated institute	E-mail address
		hans.fuchs@uk-koeln.de
)		lars.schiffmann@uk-koeln.de

ırch, educational and professional publishers, created in May 2015
ation and Springer Science+Business Media.

ıge of authorship request form - Journals (pre-acceptance)

ıse use this section to provide a new disclosure statement and, if appropriate, acknowledge any contributors who any new authors made (if applicable per the journal or book (series) policy). Please ensure these are updated in ɛpartment will not transfer the information in this form to your manuscript.

:y):

study and he made significant contribution to our
ƆAF

ırch, educational and professional publishers, created in May 2015
ation and Springer Science+Business Media.

(7)

ıge of authorship request form - Journals (pre-acceptance)

ase use this section to provide a new disclosure statement and, if appropriate, acknowledge any contributors who any new authors made (if applicable per the journal or book (series) policy). Please ensure these are updated in epartment will not transfer the information in this form to your manuscript.

:y):

study and he made significant contribution to our
DAF

W. Schroeder

ırch, educational and professional publishers, created in May 2015
ıtion and Springer Science+Business Media.

ıge of authorship request form - Journals (pre-acceptance)

ɾemoved *must* sign this declaration.
:. Please note that signatures that have been inserted as an image file are acceptable as long as it is handwritten.
ıppropriate. Delete all of the bold if you were on the original authorship list and are remaining as an author.

	Signature	Date
·oposed new authorship shown **d the addition/removal*of my thorship list** /and the proposed sponding author		
·oposed new authorship shown **d the addition/removal*of my thorship list** /and the proposed sponding author		22.04.25
·oposed new authorship shown **d the addition/removal*of my thorship list** /and the proposed sponding author		
·oposed new authorship shown **d the addition/removal*of my thorship list** /and the proposed sponding author		
·oposed new authorship shown **d the addition/removal*of my thorship list** /and the proposed sponding author		
·oposed new authorship shown **d the addition/removal*of my thorship list** /and the proposed sponding author		
·oposed new authorship shown **d the addition/removal*of my thorship list** /and the proposed sponding author		04/22/25

ırch, educational and professional publishers, created in May 2015
ation and Springer Science+Business Media.

(9)

ıge of authorship request form - Journals (pre-acceptance)

removed *must* sign this declaration.

/. Please note that signatures that have been inserted as an image file are acceptable as long as it is handwritten.

ıppropriate. Delete all of the bold if you were on the original authorship list and are remaining as an author.

	Signature	Date
·oposed new authorship shown **d the addition/removal*of my thorship list** /and the proposed sponding author	[illegible]	14.04.25
·oposed new authorship shown **d the addition/removal*of my thorship list** /and the proposed sponding author		
·oposed new authorship shown **d the addition/removal*of my thorship list** /and the proposed sponding author	[illegible]	
·oposed new authorship shown **d the addition/removal*of my thorship list** /and the proposed sponding author		
·oposed new authorship shown **d the addition/removal*of my thorship list** /and the proposed sponding author	Schmidt	15.04.25
·oposed new authorship shown **d the addition/removal*of my thorship list** /and the proposed sponding author		
·oposed new authorship shown **d the addition/removal*of my thorship list** /and the proposed sponding author		

ırch, educational and professional publishers, created in May 2015
ation and Springer Science+Business Media.

ıge of authorship request form - Journals (pre-acceptance)

	Signature	Date
·oposed new authorship shown ı **the addition/removal*of my** **thorship list** /and the proposed ;ponding author	[signature]	04/22/25
·oposed new authorship shown ı **the addition/removal*of my** **thorship list** /and the proposed ;ponding author		
·oposed new authorship shown ı **the addition/removal*of my** **thorship list** /and the proposed ;ponding author	[signature]	24.04.25

ıme		Signature	Date
	I agree to the proposed new authorship shown in section 4 **/and the addition/removal*of my name to the authorship list** /and the proposed change in corresponding author		

rst table under Section 6.

---- End of form ----

rch, educational and professional publishers, created in May 2015
ıtion and Springer Science+Business Media.

ıge of authorship request form - Journals (pre-acceptance)

	Signature	Date
oposed new authorship shown **ɟ the addition/removal*of my ŧhorship list** /and the proposed ;ponding author	[signature]	04/22/12
oposed new authorship shown **ɟ the addition/removal*of my ŧhorship list** /and the proposed ;ponding author		
oposed new authorship shown **ɟ the addition/removal*of my ŧhorship list** /and the proposed ;ponding author		

ıme		Signature	Date
	I agree to the proposed new authorship shown in section 4 **/and the addition/removal*of my name to the authorship list** /and the proposed change in corresponding author		

rst table under Section 6.

---- End of form ----

rch, educational and professional publishers, created in May 2015
tion and Springer Science+Business Media.

ıge of authorship request form - Journals (pre-acceptance)

ˈemoved *must* sign this declaration.
. Please note that signatures that have been inserted as an image file are acceptable as long as it is handwritten.
ppropriate. Delete all of the bold if you were on the original authorship list and are remaining as an author.

	Signature	Date
oposed new authorship shown **l the addition/removal*of my horship list** /and the proposed ponding author		
oposed new authorship shown **l the addition/removal*of my horship list** /and the proposed ponding author		
oposed new authorship shown **l the addition/removal*of my horship list** /and the proposed ponding author		
oposed new authorship shown **the addition/removal*of my horship list** /and the proposed oonding author		
oposed new authorship shown **the addition/removal*of my horship list** /and the proposed oonding author		
oposed new authorship shown **the addition/removal*of my horship list** /and the proposed oonding author		23.04.25
oposed new authorship shown **the addition/removal*of my horship list** /and the proposed oonding author		

:h, educational and professional publishers, created in May 2015
ion and Springer Science+Business Media.

(13)

ge of authorship request form - Journals (pre-acceptance)

	Signature	Date
ɔposed new authorship shown **the addition/removal*of my ɦorship list** /and the proposed ɔonding author		
ɔposed new authorship shown **the addition/removal*of my ɦorship list** /and the proposed ɔonding author	Schlößer	16.04.25
ɔposed new authorship shown **the addition/removal*of my ɦorship list** /and the proposed ɔonding author		

me		Signature	Date
	I agree to the proposed new authorship shown in section 4 **/and the addition/removal*of my name to the authorship list** /and the proposed change in corresponding author		

st table under Section 6.

---- End of form ----

:h, educational and professional publishers, created in May 2015
ion and Springer Science+Business Media.

ıge of authorship request form - Journals (pre-acceptance)

	Signature	Date
roposed new authorship shown **d the addition/removal*of my** **thorship list** /and the proposed sponding author		
roposed new authorship shown **d the addition/removal*of my** **thorship list** /and the proposed sponding author		
roposed new authorship shown **d the addition/removal*of my** **thorship list** /and the proposed sponding author		

ame		Signature	Date
	I agree to the proposed new authorship shown in section 4 **/and the addition/removal*of my name to the authorship list** /and the proposed change in corresponding author		

ïrst table under Section 6.

---- End of form ----

ɜrch, educational and professional publishers, created in May 2015
ation and Springer Science+Business Media.

(15)

ıge of authorship request form - Journals (pre-acceptance)

removed *must* sign this declaration.
'. Please note that signatures that have been inserted as an image file are acceptable as long as it is handwritten.
ıppropriate. **Delete all of the bold if you were on the original authorship list and are remaining as an author.**

	Signature	Date
·oposed new authorship shown **d the addition/removal*of my thorship list** /and the proposed sponding author	[illegible]	15.04.25
·oposed new authorship shown **d the addition/removal*of my thorship list** /and the proposed sponding author	[illegible]	14.04.25
·oposed new authorship shown **d the addition/removal*of my thorship list** /and the proposed sponding author		
·oposed new authorship shown **d the addition/removal*of my thorship list** /and the proposed sponding author		
roposed new authorship shown **d the addition/removal*of my thorship list** /and the proposed sponding author		
roposed new authorship shown **d the addition/removal*of my thorship list** /and the proposed sponding author		
roposed new authorship shown **d the addition/removal*of my thorship list** /and the proposed sponding author		

ırch, educational and professional publishers, created in May 2015
ation and Springer Science+Business Media.

ge of authorship request form - Journals (pre-acceptance)

emoved *must* sign this declaration.
. Please note that signatures that have been inserted as an image file are acceptable as long as it is handwritten.
ppropriate. **Delete all of the bold if you were on the original authorship list and are remaining as an author.**

	Signature	Date
oposed new authorship shown **d the addition/removal*of my thorship list** /and the proposed sponding author		
oposed new authorship shown **d the addition/removal*of my thorship list** /and the proposed sponding author		
oposed new authorship shown **d the addition/removal*of my thorship list** /and the proposed sponding author		
roposed new authorship shown **d the addition/removal*of my thorship list** /and the proposed sponding author	[illegible]	24.04.28
roposed new authorship shown **d the addition/removal*of my ıthorship list** /and the proposed sponding author		
roposed new authorship shown **ıd the addition/removal*of my ıthorship list** /and the proposed sponding author		
roposed new authorship shown **ıd the addition/removal*of my ıthorship list** /and the proposed sponding author		

arch, educational and professional publishers, created in May 2015
cation and Springer Science+Business Media.
