# Supplementary material for: The impact of postoperative atrial fibrillation on complications and mortality following Ivor Lewis esophagectomy for esophageal cancer
Source: Sci Rep. 2025 Jul 1;15:22305. doi: 10.1038/s41598-025-06239-8 (PMC12217222; doi:10.1038/s41598-025-06239-8)
Supplement: Supplementary file 3 — Supplementary Material 3 [file 41598_2025_6239_MOESM3_ESM.pdf]

Universität zu Köln

Geschäftsstelle Ethikkommission • Universität zu Köln • 50931 Köln

Anästhesieologie und operative
Intensivmedizin
Herr Dr.med Saeed Torabi
Kerpener Str. 62
50937 Köln

Per EKPool

Köln, 26.08.2024

Unser Zeichen: 24-1319-retro

Outcome-Analyse nach Ösophagusresektion: Der Einfluss von intra- und postoperativer Volumentherapie auf Mortalität und postoperative Komplikationen

Sehr geehrter Herr Dr. Torabi,

hiermit bestätigen wir, dass Ihre Anfrage zum o. g. Vorhaben am 23.08.2024 bei uns eingegangen ist.

Der Vorgang ist unter obiger Antragsnummer erfasst. Bei Nachfragen oder Korrespondenz bitten wir Sie, stets diese Antragsnummer anzugeben.

Gemäß § 15 Abs. 1 der Berufsordnung für die Nordrheinischen Ärztinnen und Ärzte besteht bei retrospektiven Vorhaben wie dem Ihrigen keine Beratungspflicht. Ferner dürfen wir auf § 6 Abs. 1 Satz 1 GDSG NRW aufmerksam machen, demzufolge das wissenschaftliche Personal die Daten, auf die es aufgrund seiner Tätigkeiten ohnehin Zugriff hat, auch wissenschaftlich nutzen darf, ohne dass hierzu das Einverständnis der betroffenen Personen notwendig wäre. Die Persönlichkeitsrechte müssen hierbei gewahrt werden.

Von daher besteht von Seiten der Ethik-Kommission **keine Notwendigkeit der Beratung, Bedenken bestehen insofern nicht.**

Medizinische Fakultät der Universität zu Köln

Geschäftsstelle der Ethikkommission

Vorsitzender
Univ.-Prof. Dr. med.
Raymond Voltz

Leitung der Geschäftsstelle
Dr. med. Guido Grass
Telefon +49 221 478 87916

Stellv. Leitung
Dipl.-Ges.-Ök. Karolina Mäder
Telefon +49 221 478 88844

Dipl.-Biol. Alice Follmann
Telefon +49 221 478 97773

Dipl.-Ges.-Ök. Christine Grimm
Telefon +49 221 478 97772

Dipl.-Ges.-Ök. Agnieszka Hompanera Torre
Telefon +49 221 478 87488

Christin Willgrod M.A.
Telefon +49 221 478 82902

Büroleitung
Barbara Ulhardt M.A.
Telefon +49 221 478 82900
Telefax +49 221 478 82905

ek-med@uni-koeln.de
www.ek-koeln.de

Servicezeiten:
Mo. – Do. 9.00 – 16.00 Uhr
Fr. 9.00 – 12.00 Uhr
und nach Vereinbarung

Besucheradresse:
Gleueler Str. 269
50937 Köln

Postanschrift:
Kerpener Str. 62
50937 Köln

Bankverbindung:
Bank für Sozialwirtschaft Köln
BLZ 370 205 00
Kto.-Nr. 8 150 000
BIC BFSWDE31

Mit freundlichen Grüßen

im Auftrag
Dipl.-Ges.-Ök. Karolina Mäder

Liste der eingereichten Unterlagen

1) Anschreiben Ethikomission ÖE Volumentherapie TorabiS.pdf vom 23.08.2024
2) Antragsbestätigung.pdf vom 23.08.2024
3) formular_retrospektive_vorhaben- VCH- ÖE- Volumentherapie-TorabiS.pdf vom 23.08.2024
4) Kurzbeschreibung.pdf vom 23.08.2024
5) nicht verfügbar - Nachweis der Registrierung als Promovend/Promovendin.pdf vom 23.08.2024
6) nicht verfügbar - Vollmacht.pdf vom 23.08.2024
7) Studienprotokoll Volumentherapie ÖE Torabis.pdf vom 23.08.2024
